# Editorial

**Published:** 1842-04-09

**Authors:** 


### THE MEDICAL EXAMINER.

### PHILADELPHIA, APRIL 9, 1842.

We observe the following curious advertisements in the London Lancet. It seems that an extensive traffic in medical degrees is driven by some of the second rate German universities, who have hit upon this lucky method of replenishing their empty coffers. Erlangen appears to be the most active in this new branch of commerce. The demand for degrees of this stamp can never arise in the United States, for the community is so cheaply supplied that the purchase of them from distant sources is unnecessary. Even in the absence of any degree, a medical pretender is at once dubbed Doctor. In England it is very different. The large mass of medical practitioners are not styled Doctor, and have no degree which authorizes it; still, if they wish a purely medical practice, the title is of some value; and as it is expensive and troublesome to obtain a diploma from a domestic institution, it may be purchased at a cheap rate in Germany. Some testimonials are of course required, however scanty, but no college which adopts this plan could look narrowly to testimonials in place of fees. Many of their graduates are really men of education, some of high acquirements, who cannot afford the time and expense necessary for the degree to which they are fairly entitled.

"Foreign Medical Association.—This Association is instituted for the purpose of counteracting the abuses of the Medical Schools of this country, by conferring Degrees in Medicine from a celebrated Continental University, on any member of the Profession who holds a regular diploma or license of practice from any College of Surgeons, Society of Apothecaries, or Chartered Medical School, in Great Britain and Ireland, without additional examination or trouble.

All communications to the Association may be addressed (pre-paid, inclosing a stamp) to Frederick Reiman, Secretary, Lovett's Coffee House, London-road, London."

"Medical Diploma.—Any gentleman wishing the Degree of M. D., at a Continental University of high repute (if he is a properly-qualified medical man,) may be greatly assisted by applying to Mr. J. George, at Mr. Eddel's, 64 Cheapside."

"Diploma in Medicine—Any properly-qualified practitioner desirous of obtaining the Degree of M. D., may, through the assistance of the advertiser, receive the same from one of the oldest Continental Universities, without absence from home. Total expense, £40.

Address, with full name and nature of qualification, to Mr. John Bond, 24 Cornhill, London."

*Professional Propriety.*—We accidentally noticed the subjoined advertisement in a late Richmond paper:—

"*Radical Cure of Strictures of the Urethra.*—Dr. ——— adopts this method of informing persons labouring under Stricture of the Urethra, that he is successfully treating that troublesome and loathsome disease upon an entirely new plan of his own invention—and that he will warrant perfect and speedy cures in every case—even the most aggravated and complex, which may be confided to his care.

☞ Consultations with Dr. ———, in cases of Stricture, by letter or otherwise, will be confidential.

——— ———, C. H., Va., Jan, 8th, 1842."

Our readers will be surprised to learn that this singular advertisement proceeds from a surgeon of considerable eminence, well known as a frequent contributor to the pages of the American Journal. Had it not come from one whose example must exert some influence upon the younger members of the profession, we should have passed it without notice. As it is, we think it the duty of journalists to express the general feeling of the profession in justly condemning such advertisements: they are, to say the least of them, entirely unprofessional.

